# Assessing the ‘active couch potato’ phenomenon in cardiac rehabilitation: rationale and study protocol

**DOI:** 10.1186/s12913-016-1313-x

**Published:** 2016-02-27

**Authors:** Nicole Freene, Borja del Pozo Cruz, Rachel Davey

**Affiliations:** Physiotherapy, Faculty of Health, University of Canberra, Canberra, ACT 2601 Australia; Department of Exercise Sciences, University of Auckland, Auckland, 1142 New Zealand; Centre for Research & Action in Public Health, Health Research Institute, University of Canberra, Canberra, ACT 2601 Australia

**Keywords:** Coronary heart disease, Sedentary behaviour, Physical activity

## Abstract

**Background:**

There is little evidence of whether or not those who have attended cardiac rehabilitation (CR) are meeting the physical activity guidelines recommended for secondary prevention of cardiovascular disease. In healthy individuals, there is evidence, that even if individuals are meeting the physical activity guidelines, the harmfulness of too much sedentary behaviour remains (active couch potato (ACP) phenomenon). Currently, there appears to be no evidence of the ACP phenomenon in those attending CR. The aims of the study are to examine the level of physical activity and sedentary behaviour in those with coronary heart disease (CHD) who have attended CR, and to investigate the potential independent associations between these behaviours and cardio-metabolic health, health-related quality of life, exercise capacity, anxiety and depression.

**Methods:**

A prospective cohort study will be conducted in Australia over 12-months. Baseline data from this study will contribute to an international, multi-centre cross-sectional study (Australia, New Zealand, United States of America, South Africa, Spain, and Portugal). Adults currently enrolled in a 6-week phase II cardiac rehabilitation program with stable CHD and receiving optimal medical treatment +/− revascularisation will be recruited. Outcome measures will be taken at baseline (commence CR), 6 weeks (complete CR), 6 and 12-months. Physical activity and sedentary behaviour will be measured using accelerometry and two questionnaires (Active Australia Survey, Past-Day Adults’ Sedentary Time questionnaire). Health outcomes will include body mass index, waist-to-hip ratio, lipid profile, blood glucose level, quality-of-life (MacNew), exercise capacity (6-min walk test), anxiety and depression (Hospital Anxiety and Depression Scale).

**Discussion:**

There has been limited investigation of the physical activity levels and sedentary behaviour of individuals with CHD attending CR. There are no studies assessing the relationship of these behaviours with health outcomes over the short and medium-term. As in healthy individuals, physical activity and sedentary behaviour may have independent effects on cardiovascular risk factors in people with CHD, which may contribute to recurrent cardiovascular events. If this is so, reducing sedentary behaviour may be a feasible first-line, additional and more achievable strategy to improve the health of those with CHD, alongside traditional recommendations to increase the time spent in moderate-to-vigorous intensity physical activity.

**Trial registration:**

Australian New Zealand Clinical Trials Registry (ANZCTR): ACTRN12615000995572

## Background

Heart disease is the leading single cause of death of men and women in Australia, and is prevalent worldwide [1, 2]. It is estimated that 34 % of all heart attacks in Australia are repeat events [3]. Not only are repeat cardiac events more likely to be fatal, they result in a higher burden of disease cost than initial events, with the total economic cost of repeat cardiac events in Australia amounting to $8.4 billion in 2010 [3].

Internationally, it is recommended that all those with coronary heart disease (CHD) be offered cardiac rehabilitation (CR), a secondary prevention program. It is widely recognised that CR decreases mortality, improves risk profiles, decreases hospital admissions, increases medication adherence and improves quality of life [4–7]. CR programs are usually exercise-based, consisting of either regular exercise alone, or a combination of exercise with education and psychological support. Moderate-intensity aerobic exercise, or physical activity, is considered a core component of CR [5, 8]. CR participants are encouraged to increase their moderate-intensity aerobic physical activity slowly and gradually, aiming to accumulate a minimum of 30 min on most, or all, days of the week, throughout life [8]. However, little is known about how much physical activity these individuals undertake outside of the CR program. It is not clear whether CR participants’ are meeting the physical activity guidelines as recommended for secondary prevention, with some indication that only 8.4 min per day of moderate-to-vigorous intensity physical activity (MVPA) is achieved in the first 12 months following diagnosis of CHD [9–11].

In healthy individuals, there is evidence that even if you do enough physical activity to meet the recommendations, by sitting too much (sedentary behaviour), the harmfulness of too much sitting time remains (the ‘active couch potato’ (ACP) phenomenon) [12]. The few prospective studies that have attempted to research this phenomenon have reached the same conclusion: meeting the public health guidelines regarding physical activity (150 min moderate-to-vigorous intensity aerobic physical activity per week) may not necessarily protect against the possible harmful effects from excessive sedentary behaviour [12, 13]. It appears that the more sedentary you are, the more likely you are to die from any cause, with sedentary behaviour recently being considered an independent risk factor for cardiovascular disease and all-cause mortality [14–16]. There is also some evidence that those with cardiovascular disease or diabetes are more likely to die from any cause if they sit for too long, independent of physical activity [17]. Therefore, it is important to take into consideration sedentary behaviour when working towards secondary prevention in those with CHD, intending to prevent further cardiac events.

To our knowledge there are limited studies assessing the effect of physical activity on health outcomes in people with CHD, with no identified studies assessing the effect of sedentary behaviour [18]. The general aim of the current study is two-fold: a) to assess the physical activity levels and sedentary behaviour (ACP phenomenon) outside the clinic in participants attending a hospital-based phase II CR program over 12 months, b) to assess the potential, independent risk of this behaviour on the cardio-metabolic health, health-related quality of life, exercise capacity, anxiety and depression of CR participants.

## Methods

### Design

A prospective cohort study will be conducted in an Australian hospital-based phase II CR program over 12-months. Baseline data from this study will contribute to an international, multi-centre cross-sectional study (Australia, New Zealand, United States of America, South Africa, Spain, and Portugal). At baseline, participants will be assessed within the first 2 weeks after enrolment in the phase II CR program. In Australia, participants will be assessed again within 2 weeks of finishing the 6-week phase II CR program, and at 6 and 12-months following admission into the phase II CR program. The phase II cardiac CR program included in this study is typical of most contemporary CR programs in Australia. The CR program is multidisciplinary, time-limited (twice a week, 6-weeks), conducted in groups, hospital-based, and has educational and supervised exercise components (one hour education plus one hour exercise) [4].

### Participants

Participants will be eligible to take part in the study if they are aged 18+ and are currently enrolled in the hospital-based phase II cardiac rehabilitation program. Participants will be included if they have stable CHD and are receiving optimal medical treatment +/− revascularisation, that is, coronary artery bypass graft surgery, percutaneous transluminal coronary angioplasty or another transcatheter procedure, or have had a myocardial infarction. Participants will be excluded if they have New York Heart Association class II-IV symptoms of heart failure (or documented signs and symptoms of chronic heart failure, with ejection fraction < 45 %), uncontrolled arrhythmias, severe chronic obstructive pulmonary disease, uncontrolled hypertension, symptomatic peripheral artery disease, unstable angina, uncontrolled diabetes, are unable to perform a submaximal walking test (6-min walk test), or are unable to wear an accelerometer due to disability, for example, if they are confined to a wheelchair. Eligible participants must have adequate English language and cognitive skills.

### Recruitment

**Fig. 1:**
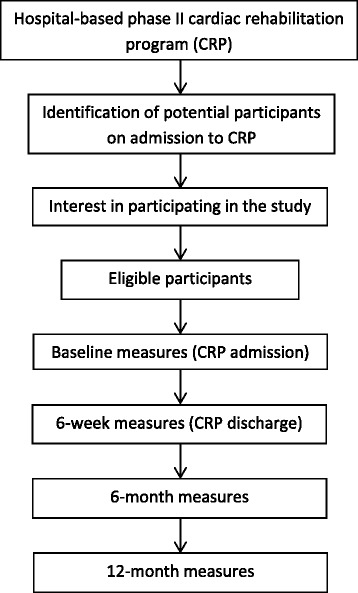
Flow of participants through the trial

CR nursing staff will recruit consecutive participants for this study within CR sessions. CR Staff will identify potential participants on admission to the phase II CR program and will provide them with details of the study. If potential participants are interested in taking part in the study, CR nursing staff will determine whether they are eligible according to the study inclusion and exclusion criteria, and gain participants’ consent. The flow of participants through the trial is illustrated in Fig. 1. The principal investigator (PI) will visit the CR team prior to participant recruitment and explain the study protocol to all team members. The PI will remain in contact with eligible participants and organise 6 and 12-month data collection to maintain participant retention. If participants are lost to follow-up, any data that has been collected will be used in the data analysis.

### Outcome measures

All study measures will be collected at baseline, 6-weeks, 6 and 12 months. All assessments will be conducted at the hospital where the CR program takes place, and will be carried out by a trained health professional. This excludes the blood samples, which will be collected at pathology collection centres. The PI will ensure that all assessors comply with data collection protocols to promote data quality and minimise measurement error.

The main outcome measures are sedentary behaviour and physical activity levels objectively measured using an accelerometer. Subjective measures of sedentary behaviour (Past-Day Adults’ Sedentary Time questionnaire, PAST) and physical activity (Active Australia Survey, AAS) have also been included in this study as they are commonly employed as outcome measures in the clinical setting due to their low cost and ease of use [19]. Haskell also suggests that physical activity data is best interpreted by adopting a combination of objective and subjective measures [20]. Dependent variables include body mass index (BMI), waist-to-hip ratio (WHR), blood lipid and glucose levels, exercise capacity (6-min walk test, 6MWT), quality of life (MacNew Heart Disease Health-related Quality of Life Questionnaire, MacNew) and anxiety and depression (Hospital Anxiety and Depression Scale, HADS).

#### Sedentary behaviour and physical activity levels

Self-reported sedentary behaviour will be assessed using the PAST [21]. The PAST is a seven-item instrument that uses past-day recall of sedentary time. It asks about time spent sitting or lying (while awake) on the previous day with questions about time spent sitting or lying while at work, travelling, watching television, using the computer (excluding work), reading (excluding work), hobbies, and any other purposes not reported in the previous items. The PAST questionnaire has been found to have acceptable reliability and validity [21].

Self-reported physical activity will be assessed using the AAS [22]. The AAS has been designed to measure participation in leisure time physical activity and to assess the participant’s knowledge of current public health messages about the health benefits of physical activity. It offers a short and reliable set of questions and applies to 1 week preceding the interview, including walking for transport. The AAS has been reported as reliable and of acceptable validity [23, 24].

A triaxial commercial accelerometer (ActiGraph GT3X, Fort Walton Beach, FL) will be used to objectively assess sedentary behaviour and physical activity. Accelerometers allow an objective measurement of quantity and intensity of movement and have been found to be reliable and valid [25]. Participants will be asked to wear the monitor on their right hip during a typical week for 24 h per day for 7 consecutive days. The raw data collected by the accelerometer, counts, will be used to obtain the time spent in different physical activity intensities [26]. We will use the Freedson Combination energy expenditure algorithm to determine the intensity cut-points [27]. Sedentary behaviour will be defined as ≤ 100 counts per minute. This outcome variable will be used to investigate whether participants have reached the World Health Organisation physical activity guidelines, and to calculate sedentary time.

Both the PAST and the AAS questionnaires will be self-administered, and will be completed at the end of the 7-day accelerometer wear time to cover the same time period.

#### Anthropometric characteristics

Height (m), weight (kg) and BMI (kg/m^2^) will be recorded using a calibrated set of scales and a stadiometer. Waist circumference and hip circumferences will be measured in centimetres using a tape measure.

#### Cardio-metabolic health

Fasting blood samples will be taken from participants and will be analysed using the Abbott Architect Ci16200 system, according to the manufacturer’s guidelines. Each sample will be assessed for a lipid standard panel (triglycerides, total cholesterol, high-density lipoprotein- HDL and non-HDL) and glucose level. Low-density lipoprotein will be determined using the Friedewald formula [28]. Blood pressure levels will be obtained using a mercury sphygmomanometer on the right arm of seated subjects.

#### Exercise capacity

The 6MWT is a commonly used objective measure of functional exercise capacity in cardiac rehabilitation [29]. The distance an individual is able to walk along a flat 25–30 m walkway over a 6 min period, with breaks as required, is recorded. The test is a self-paced, submaximal test of exercise capacity, and has been found to have a moderate-to-high reliability and validity [29].

A hospital-based exercise physiologist will perform these assessments, monitoring heart rate, oxygen saturations, the modified Borg rating of perceived exertion scale, signs and symptoms throughout. The test may be terminated by the participant or by the assessor if criteria for termination of exercise testing are met. Only one trial will be administered.

#### Health-related quality of life

The MacNew will be used for the assessment of heart disease specific health-related quality of life [30]. The MacNew is self-administered and consists of 27 items which fall into three domains (a 13-item physical limitations domain scale, a 14-item emotional function domain scale, and a 13-item social function domain scale). There are 5 items that inquire about symptoms: angina/chest pain, shortness of breath, fatigue, dizziness, and aching legs. The time frame for the MacNew is the previous two weeks, and it has good reliability and validity internationally [30].

#### Anxiety and depression

The HADS will be used for the assessment of anxiety and depression [31]. This questionnaire is a 14‐item self‐reporting questionnaire comprised of 4‐point Likert‐scaled items covering the occurrence of symptoms of anxiety (HADS‐A) and depression (HADS‐D) over the past 2 weeks. Each item on the questionnaire is scored from 0–3, so that a person can score between 0 (best outcome) and 21 (worst outcome) for either anxiety or depression.

#### Demographic and clinical questionnaire

Participants will be assessed on their socio-demographic variables (i.e. gender, age, education level, relationship status, current employment status) as well as clinical predictor variables (i.e. cardiac-related medication, other medical conditions, health care utilisation).

Eligible CR participants’ will be encouraged to adhere to the CR program, with no additional encouragement to change their physical activity and/or sedentary behaviour provided over the 12-month data collection period.

### Sample size

The G*Power software v.3.1.9.2 was used to *a priori* determine the required sample size. Previous recommendations for sample size estimation in Multiple Linear Regression problems were used [32]. With a desired effect size of f^2^ = 0.5 (large), given a maximum of 10 predictor variables including potential confounders, a sample size of *n* = 59 is needed to achieve a power of 0.95 in a test based on α = 0.05. Assuming a dropout rate of 25 %, 75 participants will be recruited for the Australian study.

### Statistical analysis

The number of minutes per day spent in sedentary behaviour will be calculated using standard count-based intensity threshold values of counts per minute: < 100 for sedentary behaviour (<1.5 MET). Four further summary measures of sedentary behaviours will calculated per day and averaged over valid days: (a) percentage of the wear-day spent in sedentary behaviour (b) number of sedentary bouts (defined as a period of consecutive minutes where the accelerometer registered <100 counts/minutes) (c) average duration of sedentary bouts and (d) number of sedentary breaks (defined as at least 1 min where the accelerometer registered ≥ 100 counts/minute following a sedentary bout). The Freedson Combination energy expenditure algorithm will be used to determine the intensity cut-points for MVPA. This outcome variable will be used to investigate whether participants have reached the World Health Organisation physical activity guidelines.

Linear regression models will be performed at each data collection point, where the dependent variables will be each of the health variables available. The models will have the different summary measures of sedentary behaviour (i.e. one model will be performed with each one) as main independent variables, and models will also be performed for MVPA and each of the different physical activity intensities (i.e. light, moderate and vigorous physical activity). If it is the case, quadratic trends might be explored. The models will be adjusted for total time of activity (i.e. counts per min ≥ 100), gender, socioeconomics variables, BMI and other potential confounders available.

Additional analyses will be completed with a one-way repeated measures analysis of variance (ANOVA) to test for differences in physical activity and sedentary behaviour (with a 95 % confidence interval (CI)) within the Australian sample over 12 months. Intention-to-treat analysis will be used at 6-weeks, 6 and 12-months where data is missing, bringing the last value forward. A maximum of three attempts will be made to contact participants so outcome measures can be obtained, making the analysis more complete.

### Ethical approval

This study was approved by the ACT Health Human Research Ethics Committee in August 2015 (Project reference: ETH.5.15.076).

### Trial status

Study protocol.

## Discussion

Over 12 months we will assess the physical activity, sedentary behaviour levels and health outcomes of those with CHD attending a hospital-based phase II CR program. Specifically, we will measure the physical activity and sedentary behaviour levels of participants’ with CHD at the start of a phase II CR program, at the end of the program, and at 6 and 12-months to determine the short and medium-term changes in physical activity and sedentary behaviour levels. Few studies have determined whether those attending CR achieve the public health physical activity guidelines, as recommended, over the short and medium term, and no known studies have reported on CR participants’ sedentary behaviour, or sitting time. Importantly, we will also investigate the relationships between physical activity, sedentary behaviour and health outcomes at each point-in-time to determine the potential, independent risk of these behaviours, including the potential for the ACP phenomenon in CR participants.

One of the strengths of the trial is the pooling of baseline results from multiple sites, internationally. Combining data will increase the strength of statistical power analyses, with the sample size to be recruited providing adequate statistical power to detect relationships between the variables. The Australian single cohort study is prospective, allowing all the relevant information to be collected, including the objective and subjective measurement of physical activity and sedentary behaviour. The outcome measures are also collected in a ‘real-world’ situation, reflecting common practice for CR in Australia.

Limitations to the study include multiple assessors of outcome measures despite assessors remaining consistent for each outcome measure, for example, exercise capacity will always be assessed by an exercise physiologist. There is also a lack of randomisation, with recruitment of participants occurring consecutively. Other possible limitations include the potential for loss of participants to follow-up, and few participants achieving the outcome of interest, the ACP phenomenon.

One third of all heart attacks in Australia are repeat events, they are more likely to result in death and are costly. If sedentary behaviour is found to be an important consideration for secondary prevention of CHD, reducing sedentary behaviour, or sitting time, may be a feasible additional or first-line strategy to improve the health of those with CHD, alongside traditional recommendations to increase the time spent in moderate-to-vigorous intensity physical activity. Therefore, the outcomes from this study may have implications for CR research, policy and practice, and ultimately improve the health of those with CHD.
